# Performance measures for in-hospital care of acute ischemic stroke in public hospitals in Chile

**DOI:** 10.1186/1471-2377-13-23

**Published:** 2013-03-06

**Authors:** Lorena Hoffmeister, Pablo M Lavados, Merce Comas, Carolina Vidal, Rodrigo Cabello, Xavier Castells

**Affiliations:** 1 School of Public Health, Facultad de Medicina, Universidad Mayor, Santiago, Chile; 2 Neurology Service, Clínica Alemana de Santiago, Universidad del Desarrollo, Santiago, Chile; 3 Department of Neurological Sciences, Facultad de Medicina, Universidad de Chile, Santiago, Chile; 4 Department of Epidemiology and Evaluation, Hospital del Mar, Barcelona; IMIM (Hospital del Mar Medical Research Institute), Barcelona; Red de Investigación en Servicios de Salud en Enfermedades Crónicas (REDISSEC), Mar Teaching Hospital, 25-29 Passeig Marítim, Barcelona, 08003, Spain

**Keywords:** Acute ischemic stroke, Stroke care, Thrombolysis

## Abstract

**Background:**

The aim of this study were to describe acute care of ischemic stroke patients and adherence to performance measures, as well as the outcomes of these events, in a sample of patients treated in public hospitals in Chile.

**Methods:**

We retrospectively reviewed the medical charts of patients with ischemic stroke from a sample of seven public hospitals in the Metropolitan Region of Santiago. We analyzed adherence to the following evidence-based measures: clinical evaluation at admission, use of intravenous thrombolysis, dysphagia screening and prescription of antithrombotic therapy at discharge. As outcome measures we analyzed post-stroke pneumonia and 30-day case-fatality. We used a logistic regression model by each outcome with generalized estimating equations, which accounted for clustering of patients within hospitals and included sex, age (years), clinical status at admission (reduced level of consciousness, speech disturbance, aphasia and hemiplegia), comorbidities, dysphagia screening and neurological evaluation at admission as measures of acute stroke care.

**Results:**

We reviewed the charts of 677 patients, of which 52.3% were men. The mean age was 69.8 years in women and 66.3 years in men. Diagnosis of stroke was confirmed by a computed tomography scan within 4.5 hours of symptom onset in only 9.6% of the patients. Intravenous thrombolysis was administered in 1.7%. Dysphagia screening was performed in 12.1% (95% CI 9.7-15.0) and antithrombotic therapy was prescribed in 68.9% (95% CI 64.6-72.9). Pneumonia was diagnosed in 23.6% (95% CI 20.4-27.2). Thirty-day fatality was 8.7% (95% CI 6.7-11.3). The variables independently associated with 30-day case fatality were age (OR 1.08, 95% 1.06-1.10), pneumonia (OR 7.7, 95% 95% CI 4.0-14.7), aphasia (OR 2.4, 95% CI 1.1-5.6), reduced level of consciousness (OR 2.4, 95% CI 1.3-4.4), and speech disturbance (OR 1.4, 95% CI 1.0-1.9). No association was found between 30-day case fatality and dysphagia screening or neurological evaluation at admission. The factors associated with post-stroke pneumonia were female sex (OR 1.6, 95% CI 1.0-2.3), age (OR 1.04 95% CI 1.03-1.05), diagnosis of diabetes (OR 1.8, 95% CI 1.4-2.4), aphasia (OR 2.0, 95% CI 1.5-2.7), hemiplegia (OR 1.6, 95% CI 1.1-2.4), and reduced level of consciousness on admission (OR 3.4, 95% CI 2.1-5.5). No association was found between pneumonia and dysphagia screening or neurological evaluation at admission.

**Conclusions:**

Adherence to evidence-based performance measures was low. Administration of intravenous thrombolysis was particularly low and diagnostic confirmation of ischemic stroke was delayed. The occurrence of post-stroke pneumonia was frequent and should be reduced. To improve acute stroke care in Chile, organizational change in the health service is urgently needed.

## Background

The global burden of stroke is greater in middle and low-income than in high-income countries [1,2]. In Chile, the annual adjusted incidence of stroke is 97.4 per 100,000 inhabitants [3], and these events cause 9% of all deaths. In the population aged 45–84 years, 65% of strokes are ischemic, corresponding to an incidence rate for a first event of 59.6 per 100,000 inhabitants, with a mean age of 68.6 years in women and 64.7 in men [4]. Only a few interventions have been demonstrated to improve prognosis after an acute ischemic stroke. Such interventions include admission to an organized Stroke Unit, which reduces death and disability and cost in all types of stroke irrespective of age or sex [5], and enhancing adherence to important clinical practice guideline recommendations [6]. The chance of a good outcome at 6 months is increased by aspirin administration within 48 hours of symptom onset, while, in large hemispheric infarctions, 3-month mortality is decreased by hemicraniectomy within 48 hours of symptom onset to prevent brain herniation [7].

The only specific drug treatment with proven cost-effectiveness in reducing the disability caused by ischemic stroke is intravenous thrombolysis with recombinant tissue plasminogen activator (rtPA) [8,9]. The effectiveness of this procedure decreases according to the interval between stroke onset and the start of treatment, which is not recommended after 270 minutes [10,11]. National [12] and international clinical guidelines recommend the use of thrombolysis in the acute management of ischemic stroke [9,13,14]. Although these recommendations were made several years ago, studies in high-income countries have reported low use of intravenous thrombolysis, even among patients admitted to hospital within the therapeutic window [15]. In Australia, intravenous thrombolysis is used in 7% of all patients with ischemic stroke, mainly in hospitals with stroke units [16]. A study performed in the USA found that intravenous thrombolysis was used in 2.4% of admissions for ischemic stroke [17]. Isolated publications in low-middle income countries have reported that the use of this treatment is even lower [18].

The systematic use of clinical guidelines depends largely on health service organization. In the USA, the use of intravenous thrombolysis and other evidence-based performance measures has significantly improved in hospitals adhering to the *Get with the Guidelines-Stroke* program [19,20]. In Spain, a Stroke Program audit of the quality of in-hospital stroke care reported significant improvements in preventive measures for concomitant conditions or potential complications, as well as in the early evaluation of the need for care [21]. After stroke services were redesigned in Australia, brain imaging within 24 hours after stroke and prescription of anti-thrombotic therapy at discharge significantly increased [6].

In Chile, the system of Explicit Health Guarantees (EHG) for ischemic stroke was implemented in 2005, which guaranteed access to confirmation of diagnosis and acute treatment of strokes to 91.0% of the Chilean population, including clinical practice guidelines with evidence-based recommendations [12]. The development of organized Stroke Units is only now starting to happen in Chile and was not included as part of the 2005 EHG for ischemic stroke.

The aim of this study was to assess acute care of ischemic stroke and adherence to performance measures, as well as to identify the outcome of these events, in a sample of patients treated in public hospitals in Chile from 2007 to 2009.

## Methods

We retrospectively reviewed the medical charts of a sample of patients aged 15 years and above, hospitalized with a diagnosis of ischemic stroke in 7 out of 22 public hospitals of the Metropolitan Region of Santiago, Chile. Overall, the selected hospitals provide health care to an adult population of 5,375,908 persons, representing 40.4% of the Chilean population. In 2009, these hospitals received 85.3% of all admissions for stroke in the Metropolitan Region of Santiago and 55.3% of those in the country as a whole, admitted 150 to 878 patients per year. These hospitals are referral centers for stroke care in each of the six territorially organized health services of the public health care network in the Metropolitan Region of Santiago. All study hospitals had adult emergency services and access to on-site computed tomography (CT) 24 hours per day and 365 days per year. We excluded pediatric hospitals (n = 5), subspecialty institutes (n = 7), hospitals without emergency departments (n = 3), and community hospitals without CT scanners (n = 8). The sampling frame was the Management System of the Explicit Health Guarantees, which is mandated to routinely register all patients with ischemic stroke admitted to hospitals in the public sector. All patients with a main admission diagnosis of ischemic stroke (ICD-10 I63) between August 2007 and August 2009 were included. The patients were identified through their national identification number, sex, age at event, and the admitting hospital. The study protocol was approved by the Ethics Committee of the Faculty of Medicine of the *Universidad Mayor*, Santiago, Chile.

### Data collection

A structured case report form was designed and tested in three hospitals in the Metropolitan Region of Santiago. Data were extracted by a team of researchers external to the selected hospitals, composed of clinical nurses with experience in adult emergency departments. All nurses were trained in the protocol and calibration was performed. If the measures were not documented, they were considered not to have been performed.

To check the quality of data collection, an external audit was carried out by blinded and independent researchers. This audit consisted of double data collection of approximately 10% of the patients admitted to each hospital and selected randomly from the patient lists. To determine consistency among raters, interrater reliability was analyzed using the Kappa statistic.

### Study variables

The variables were selected based on a review of the literature on the acute management of ischemic stroke, evidence-based recommendations in protocols and clinical practice guidelines, expert evaluation (neurologists, clinical epidemiologists and emergency department nurses), and the availability of data in the medical chart. We chose the performance measures that we believed to be most indicative of the outcomes of case fatality and pneumonia. Adherence to the following performance measures was included: a) completeness of the data and time from symptom onset to CT confirmation of the ischemic stroke diagnosis by neurologist, b) the time interval from symptom onset to CT scan and diagnostic confirmation of ischemic stroke by neurologist, c) the percentage of patients who underwent dysphagia screening through a simple, valid bedside test before receiving any food, fluids or medication and within 48 hours of hospital admission (excluding comatose patients), d) the proportion of patients with neurological evaluation on admission through the Glasgow Coma Scale [22], 3) the proportion of ischemic stroke patients with prescribed antithrombotic therapy at hospital discharge, and f) the percentage of patients administered intravenous thrombolysis. We also included the occurrence of post-stroke pneumonia diagnosed after hospital admission, and 30-day case fatality. Pneumonia was defined as pneumonia occurring during hospitalization, documented by a physician, and requiring antibiotic treatment.

Because patients can die after hospital discharge, survival was determined through the link between the variables identifying ischemic stroke patients (complete name and unique identity number) and the official mortality register. This register is exhaustive and the cause of death was medically certified in 99.3% of deaths in 2007, while 2.9% of deaths were classified as poorly defined [23]. The death coding process is carried out according to standardized and centralized criteria in the Statistics and Health Information Department of the Ministry of Health and the main cause of death is coded according to the ICD-10 classification.

### Statistical analysis

The sample size was calculated to provide an estimated 15.0% prevalence of in-hospital pneumonia, 2.5% precision, a 95% confidence interval, and 12% predicted losses. A population of 10,467 ischemic stroke patients in the Metropolitan Region of Santiago was used to estimate the sample. The overall sample consisted of 821 patients. Due to differences in the volume of patients admitted to the study hospitals, stratified random sampling by hospital (stratum) was used to select ischemic stroke patients from the list of patients. In each hospital, an equal number of patients (n = 117) were randomly sampled, except in one hospital where 119 patients were selected. Thus, the probability of including a patient in the sample depended on the hospital’s size. According to the sample design and to obtain representative results, sampling weights were calculated as the inverse probability of being included in the study. Descriptive results were weighted.

Performance measures were calculated by taking the number of patients receiving a particular intervention as the numerator, and the total number of patients eligible for this intervention as the denominator. Proportions and 95% confidence intervals were calculated for the population characteristics and for the study variables. Proportions were compared among hospitals by using the Kruskal-Wallis test.

To estimate the adjusted odds ratios of 30-day fatality and in-hospital pneumonia according to adherence to selected performance measures, dysphagia screening and neurological evaluation, we used multivariable logistic regression with generalized estimating equations (GEE), which accounted for the correlations among patients within hospitals. The model for 30-days fatality was adjusted by the patients’ demographic characteristics (sex and age at event), comorbidities (diabetes and hypertension), in-hospital pneumonia (because it is an important predictor of post-stroke fatality) [24] and clinical status at admission (aphasia, hemiplegia, reduced level of consciousness, and speech disturbance). These variables showed the degree of neurological impairment assessed within the first few hours after arrival at the hospital and were included in the regression model as proxies of stroke severity [25]. The model for post-stroke pneumonia was adjusted using the same patient’s characteristics, and excluding pneumonia. A p-value of < 0.05 was considered statistically significant and odds ratios with their 95% CI were calculated.

Potential selection biases were assessed by comparing the distributions of sex, mean age, and the proportion of 30-day fatality among participants and non-participants in the study. The Chi-squared test was used to compare differences in sex and 30-day fatality, and Student’s *t*-test was used to compare age.

All statistical analyses were performed using the Statistical Package for the Social Sciences (SPSS) program, version 20.0.

## Results

**Figure 1:**
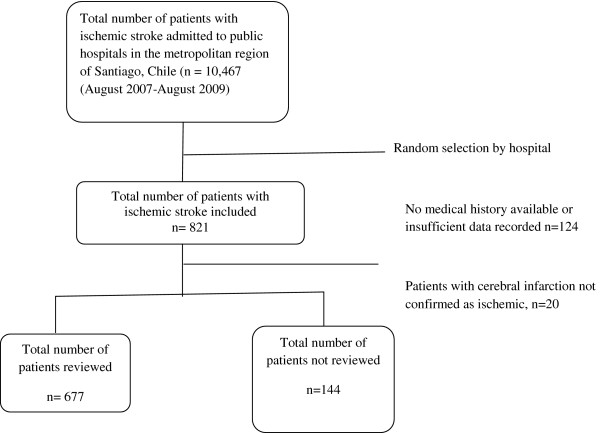
Flow chart of patient inclusion in the study.

Of 821 patients selected, 677 were included in the study. We excluded 124 patients because their medical charts could not be found after five searches on different dates or because the charts contained insufficient information for diagnosis of an ischemic stroke (Figure 1). Twenty patients without confirmed ischemic stroke were also excluded. The overall interrater agreement was 0.75, and more than 85% of the variables achieved kappa values above 0.6 (using the Landis & Koch classification) [26]. The number of excluded patients was significantly greater in hospital 2 than in the remaining hospitals. Overall, there were no significant differences between participants and non-participants in sex distribution (p-value: 0.84), mean age (p-value: 0.60), or 30-day fatality (p-value: 0.27).

Men accounted for 52.3% of the sample (Table 1). The mean age was 69.8 years (95% CI 68.0- 71.6) in women and 66.3 years (95% CI 64.9- 67.6) in men. A history of hypertension was found in 77.0% and a diagnosis of diabetes mellitus in 28.5%. There were no significant differences among hospitals in patient characteristics (Table 2).

**Table 1:** Characteristics of the study population

	**n**	**Percentage (95% CI)**
Sex		
Male	359	52.3 (48.2-56.4)
Female	318	47.7 (43.6-51.8)
Age		
<=50 years	82	12.6 (10.1-15.7)
51 - 60 years	91	13.9 (11.3-17.0)
61 - 70 years	180	26.4 (23.0-30.2)
71- 80 years	206	30.2 (26.5-34.1)
More than 80 years	114	16.9 (14.0-20.2)
Comorbidities		
Diagnosis of diabetes	197	28.5 (25.0-32.3)
History of hypertension	533	77.0 (73.3-80.4)
Hospitals		
Hospital 1	99	25.5
Hospital 2	64	8.6
Hospital 3	110	13.0
Hospital 4	98	3.8
Hospital 5	98	12.6
Hospital 6	105	18.9
Hospital 7	103	17.7

**Table 2:** Characteristics of the study population by hospital

		**Hospital 1**			**Hospital 2**			**Hospital 3**			**Hospital 4**			**Hospital 5**			**Hospital 6**			**Hospital 7**			**Kruskall-Wallis p-value**
		**%**	**CI (95%)**		**%**	**CI (95%)**		**%**	**CI (95%)**		**%**	**CI (95%)**		**%**	**CI (95%)**		**%**	**CI (95%)**		**%**	**CI (95%)**		
			**Lower**	**Upper**		**Lower**	**Upper**		**Lower**	**Upper**		**Lower**	**Upper**		**Lower**	**Upper**		**Lower**	**Upper**		**Lower**	**Upper**	
Sex	Men	47.5	37.7	57.5	57.8	45.1	69.5	47.3	38.1	56.8	55.1	45.0	64.8	58.2	48.0	67.7	57.1	47.4	66.4	50.5	40.8	60.2	0.487
	Women	52.5	42.5	62.3	42.2	30.5	54.9	52.7	43.2	61.9	44.9	35.2	55.0	41.8	32.3	52.0	42.9	33.6	52.6	49.5	39.8	59.2	
	Total	100			100			100			100			100			100			100			
Age (years)	<=50	15.8	9.7	24.7	18.8	10.8	30.5	7.3	3.6	14.0	13.3	7.8	21.7	8.2	4.1	15.7	11.4	6.5	19.2	13.6	8.1	21.8	0.219
	51 - 60	12.6	7.2	21.1	12.5	6.3	23.4	10.9	6.2	18.4	10.2	5.5	18.1	17.3	11.0	26.3	13.3	8.0	21.4	17.5	11.2	26.2	
	61 - 70	25.3	17.4	35.1	23.4	14.5	35.7	29.2	21.3	38.5	29.6	21.3	39.5	22.4	15.2	31.9	31.4	23.2	41.1	24.3	16.9	33.6	
	71- 80	28.4	20.1	38.5	32.8	22.2	45.5	28.2	20.4	37.4	32.7	24.0	42.7	31.6	23.1	41.6	26.7	19.0	36.1	35.0	26.2	44.8	
	80 and older	17.9	11.3	27.1	12.5	6.3	23.4	24.5	17.3	33.6	14.3	8.6	22.9	20.4	13.5	29.7	17.1	11.0	25.7	9.7	5.2	17.3	
	Total	100			100			100			100			100			100			100			
Diabetes	Without diagnostic	72.7	63.0	80.7	68.8	56.1	79.1	69.1	59.8	77.1	69.4	59.4	77.8	70.4	60.5	78.7	78.1	69.0	85.1	67.0	57.2	75.5	0.678
	With diagnostic	27.3	19.3	37.0	31.3	20.9	43.9	30.9	22.9	40.2	30.6	22.2	40.6	29.6	21.3	39.5	21.9	14.9	31.0	33.0	24.5	42.8	
	Total	100			100			100			100			100			100			100			
Hypertension	Without diagnostic	31.3	22.8	41.3	20.3	12.0	32.3	19.1	12.7	27.6	16.3	10.2	25.2	20.4	13.5	29.7	24.8	17.3	34.1	16.5	10.4	25.1	0.128
	With diagnostic	68.7	58.7	77.2	79.7	67.7	88.0	80.9	72.4	87.3	83.7	74.8	89.8	79.6	70.3	86.5	75.2	65.9	82.7	83.5	74.9	89.6	
	Total	100			100			100			100			100			100			100			

The percentage of patients with complete information on the date and time of symptom onset was 47.0% (95% CI 42.9-51.1). Significant differences were found among hospitals (p <0.05), with a range of between 36.7% and 58.1%. In 58.2% of patients (95% CI 54.2-62.2), the date and time of CT scans and diagnostic confirmation by a neurologist was recorded. Recording of the date and time of CT scans was significantly lower in two hospitals than in the remaining hospitals (p <0.05).

**Figure 2:**
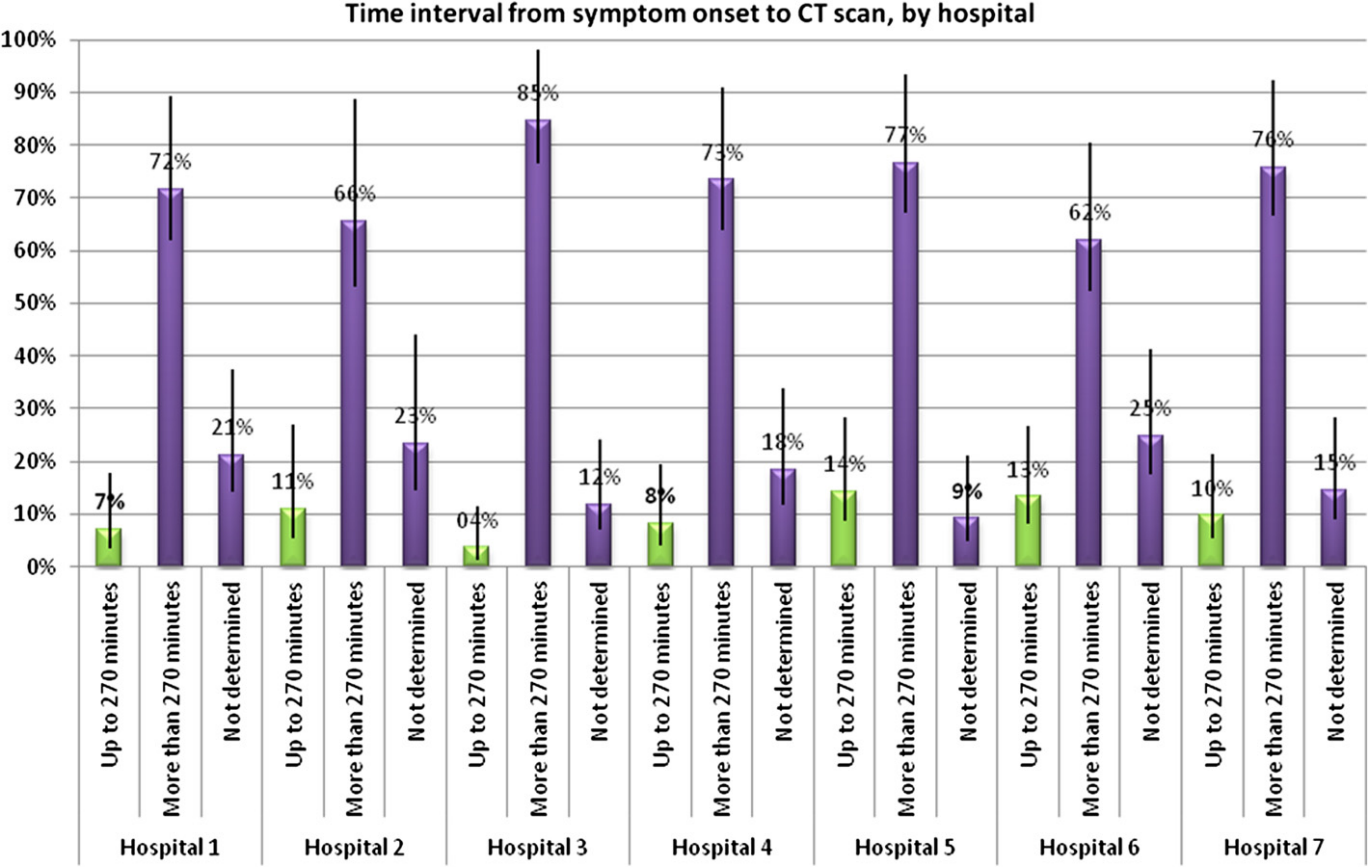
Time interval from symptom onset to CT scan by hospital.

A CT scan was performed within 24 hours of symptom onset in only 45.2% of patients, while the interval between symptom onset and CT scan could not be determined in 21.1% of the medical charts (95% CI 17.8-24.9). An interval of up to 270 minutes between symptom onset and CT-based confirmation of the diagnosis occurred in 9.6% of the patients (95% CI 7.4-12.2). This percentage varied among hospitals, ranging from 3.6% (95% CI 1.4-9.4) to 14.3% (95% CI 8.6-22.7) (Figure 2).

**Figure 3:**
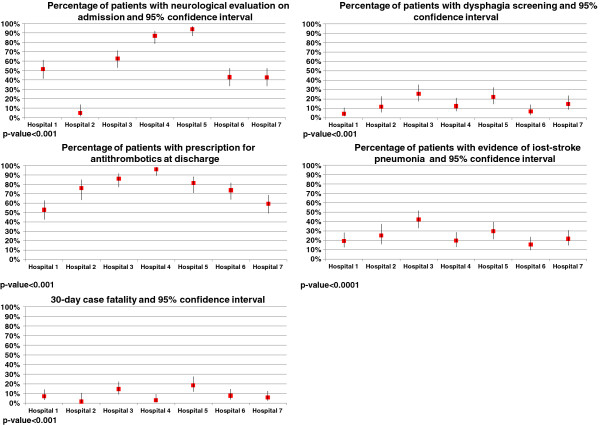
Performance and outcome measures by hospital.

Intravenous thrombolysis was used in 15 patients in only two hospitals (Table 3), representing 1.7% (95% CI 1.0-3.0). Neurological evaluation on admission using a standard clinical scale was performed in 52.4% (95% CI 48.3-56.5), with significant differences among hospitals (p = 0.03). Dysphagia screening was carried out in 12.1% (95% CI 9.7-15.0) of patients with indication for this procedure, with significant differences among hospitals, ranging from 4.2% to 25.4% (p <0.0001). Antithrombotic therapy was prescribed at discharge in 68.9% (95% CI 64.6-72.9), with significant differences among hospitals (p <0.0001). In-hospital pneumonia occurred in 23.6% (95% CI 20.4-27.2), but this percentage was much higher in hospital 3: 41.9% (95% CI 32.9-51.4) compared with the remaining hospitals (p < 0.0001). Case-fatality at 30 days was 8.7% (95% CI 6.7-11.3) with significant differences among centers (p <0.0001), hospital 5 showing the highest mortality (Figure 3).

**Table 3:** Performance measures, percentage of patients with pneumonia and 30-days case fatality

**Measure**	**Number of interventions performed/number indicated**	**% (95% CI)**
Percentage of patients with neurological evaluation on admission	389/677	52.4 (48.3-56.5)
Dysphagia screening	84/609*	12.1 (9.7-15.0)
Prescription of antithrombotics at discharge	457/612^†^	68.9 (64.6-72.9)
Intravenous thrombolysis	15/677	1.70 (1.0-3.0)
In-hospital pneumonia	167/677	23.6 (20.4- 27.2)
30-day case fatality	59/677	8.7 (6.7-11.3)

^*^68 patients who were unconscious at hospital admission were excluded.

^†^ Patients who died during their hospital stay were excluded.

The GEE for 30-day case-fatality (Table 4), which included the effect of clustering patients in hospital, showed that demographic and clinical variables independently associated with an increased probability of death were age (OR 1.08, 95% CI 1.06-1.10), post-stroke pneumonia (OR 7.65, 95% CI 3.98-14.72), and reduced level of consciousness (OR- 2.41, 95% CI 1.32-4.41) at hospital admission. No association was found between the performance of neurological evaluation (OR 2.02, 95% CI 0.77-5.30) and dysphagia screening (OR 0.52, 95% CI 0.26-1.04) and the likelihood of 30-day fatality.

**Table 4:** Factors associated with 30-day case-fatality and in-hospital pneumonia, adjusted odds ratios from generalized estimating equations

	**30-day case-fatality**		**In-hospital pneumonia**	
	**Adjusted OR (95% CI)**	**p-value**	**Adjusted OR (95% CI)**	**p-value**
Sex: female	1.48 (0.82-2.27)	0.28	1.55 (1.03-2.33)	0.03
Age (years)	1.08 (1.06-1.10)	0.00	1.04 (1.03-1.05)	0.00
Diagnosis of diabetes	0.53 (0.24-1.15)	0.11	1.79 (1.35-2.38)	0.00
Diagnosis of hypertension	1.90 (0.49-7.30)	0.35	0.82 (0.54-1.24)	0.33
Aphasia on admission	2.42 (1.05-5.61)	0.04	1.97 (1.45-2.69)	0.00
Hemiplegia on admission	0.57 (0.23-1.39)	0.21	1.60 (1.08-2.38)	0.02
Reduced level of consciousness on admission	2.41 (1.32-4.41)	0.00	3.37 (2.07-5.48)	0.00
Speech disturbance on admission	1.39 (1.02-1.90)	0.04	1.24 (0.88-1.74)	0.22
Post-stroke pneumonia	7.65 (314.72)	0.00	-	-
Neurological evaluation	2.02 (0.77-5.30)	0.15	1.07 (0.79;1.44)	0.66
Dysphagia screening	0.52 (0.26-1.04)	0.07	1.58 (0.60; 4.15)	0.36

Models adjusted by all factors in this table and clustering of patients by individual hospital.

For post-stroke pneumonia (Table 4), the demographic and clinical variables independently associated with an increased risk of pneumonia were female sex (OR 1.55, 95% CI 1.03-2.33), age (OR 1.04, 95% CI 1.03-1.05), diabetes (OR 1.79, 95% CI 1.35-2.38), aphasia (OR 1.97, 95% CI 1.45-2.69), hemiplegia (OR 1.60, 95% CI 1.08-2.38), and reduced level of consciousness (OR 3.37, CI 95% 2.07-5.48). No association was found between dysphagia screening and neurological evaluation and post-stroke pneumonia.

## Discussion

In this study, we found that adherence to evidence-based performance measures in the acute care of ischemic stroke in a sample of public hospitals in the Metropolitan Region of Santiago was very low. Several programs to improve the quality and delivery of care have prospectively investigated performance measures before and after intervention programs around the world and have described similar results before organizational interventions in acute stroke care [20,21,27-30].

In our study, a CT scan was performed within 24 hours of symptom onset in less than 50% of patients and within 4.5 hours in less than 10%, limiting the possibility of acute care, especially intravenous thrombolysis or hemicraniectomy. The literature reveals considerable variability in arrival times to hospital. In the Paul Coverdell Registry, the median time from hospital arrival to brain imaging among all patients was 1 hour, 12 minutes (mean: 2 hours, 36 minutes) [27], while in Korea the median time interval from symptom onset to hospital arrival was 7 hours, 54 minutes [31]. Consistent with the finding of delays from symptom onset to CT confirmation of diagnosis, intravenous thrombolysis was administered in only 1.7% of the patients, a lower percentage than that in high-income countries [16,19], and similar to that reported in Argentina and China, where 1.05% and 2.4% of patients with acute ischemic stroke were treated with intravenous thrombolysis [30,32]. This low use was to be expected, since systematic organizational changes have not been implemented in hospitals and thrombolysis is usually administered in hospitals with stroke units [16].

We detected substantial deficiencies in the thoroughness and accuracy of registration of the times of symptom onset and CT confirmation of diagnosis. Monitoring the time from symptom onset to the distinct interventions is essential to improve patient care and adherence to evidence-based performance measures in the acute management of stroke and to reduce pre-admission and post-admission barriers to treatment [15]. Of 56,969 patients in the Paul Coverdell Registry, the time from symptom onset to hospital arrival was unknown or not recorded in 57.8% [27].

This study shows that clinical neurological evaluation measures such as the National Institute of Health Stroke Scale (NIHSS) have not been incorporated in routine clinical practice in the emergency departments of the hospitals in the sample. The NIHSS has established validity and reliability for use in the clinical evaluation of stroke patients and is strongly predictive of early functional recovery and long-term outcome. This scale can be administered by physicians, research workers, and nurses alike and has proven intra- and inter-rater reliability [33]. In addition to the low use of the NIHSS, we also found that early evaluation of neurological deficit was not systematically performed in all the hospitals studied.

Dysphagia screening was particularly low in our study and could strongly influence the incidence of pneumonia. In the Canadian Stroke Network Registry, dysphagia screening was performed in 56% of patients [28]. A study using the Paul Coverdell Registry showed that unscreened patients were at a higher risk of pneumonia than screened patients [34].

In the present study, more than one-fifth of the patients developed in-hospital pneumonia. Post-stroke pneumonia is a potentially preventable complication that is associated with poor outcome [35]. In the multivariable analysis, the factors associated with pneumonia were age, female sex, a reduced level of consciousness on admission, aphasia, hemiplegia and a diagnosis of diabetes. Neither dysphagia screening nor clinical evaluation care contributed significantly to post-stroke pneumonia. Dysphagia screening was carried out in a very small proportion of patients and could not fully be evaluated in the multivariable model. Clinical evaluation on admission, as defined in current clinical practice, was insufficiently specific to identify other variables with proven prognostic value in the risk of post-stroke pneumonia, such as pre-existing dependency, non-lacunar vs lacunar stroke, chronic obstructive pulmonary disease and other factors [36]. Finlayson reported that health care determinants, such as stroke unit admission did not predict the occurrence of pneumonia but were, however, associated with decreased mortality from this infection, indicating the need to intensify acute stroke care [36]. Like Aslayen et al. [37], we found an increased risk of pneumonia in women and patients with diabetes, which could be explained by the higher risk of infections in diabetic patients.

Among secondary prevention measures, compliance with recommendations on the prescription of antithrombotic agents at discharge was lower than expected and these drugs were not prescribed in a third of the eligible patients; variations among hospitals were also found. This finding is particularly worrisome as prescription of these drugs at discharge is one of the most widely used and cheapest secondary preventive measures. In most registries, compliance with this performance measure was usually above 90% in both high- and low-medium income countries [20,21,30]. Likewise, no record was found in the medical charts of disability evaluation at discharge or the need for community-based rehabilitation. Although not usually considered performance measures of acute stroke care, both measures are associated with improved outcomes [38].

The case fatality rates in our study are similar to the mean case fatality rates reported in countries belonging to the Organization for Economic Cooperation and Developmet (OECD) [39]. Like other studies [35], we found that the factors that contributed to explaining the likelihood of death at 30 days were pneumonia, age [36], speech disturbance, reduced level of consciousness and aphasia. Nevertheless, the two interventions selected did not have a significant effect on this outcome.

Our study has several limitations that could bias the results. We were unable to retrieve 17.5% of the sample because of the poor quality of medical registration in the patients’ charts and the impossibility of retrieving the charts in one hospital after the 2010 earthquake. However, no difference was found by demographic characteristics and 30-day fatality between patients included in the sample and those not included. Therefore, we believe that the probability of selection bias is small and that our results reflect the reality of stroke care in these hospitals. Nevertheless, they may not reflect the care in hospitals in more isolated and less populated regions of the country, where standards could be lower and outcomes poorer, as shown in rural Australia by Cadilhac et al. [40].

Another limitation of this study is that we could not measure the effect of organized patient care (stroke units) as these facilities are only recently being implemented in some public hospitals in the Metropolitan Region of Santiago. In addition, we had no standardised summary measure of stroke severity such as the NIHSS and relied on other objective clinical measures at time of admission to account for stroke severity in our statistical models.

A strength of this study is that we were able to obtain data from hospitals admitting more that 50% of all patients with ischemic stroke in the country. We believe the findings of this study have good external validity for Chile as the age and sex distribution of the patients included was similar to those of the patients in the only community-based stroke study published to date the PISCIS study [3].

## Conclusions

This is the first study to reveal the low implementation and adherence to evidence-based performance measures in hospitals admitting more than 50% of all ischemic stroke patients in Chile. These measures are part of the National Chilean Clinical Practice Guidelines for stroke, and reimbursement is guaranteed by health insurance agencies for some recommendations such as the use of CT scanning and thrombolytic therapy. We hope that these results will encourage decision makers to implement surveillance registries and modify the organization of health services that provide care to patients with stroke; in other countries, such actions have shown promising results in a relatively short term [6,19,21]. Furthermore, to increase the possibility of hyperacute interventions such as thrombolysis in a larger population, programs directed at lowering the barriers to prompt diagnosis and arrival at emergency departments should be designed and implemented.

## Competing interests

The authors have no conflicts of interest.

## Authors’ contributions

LH made substantial contributions to the conception and design of the study, data collection and analysis, interpretation of the results and drafting of the manuscript. PL substantially contributed to the conception and design of the study, analysis and interpretation of the results and drafting of the manuscript. CV performed the data analysis, participated in data collection, interpretation and preparation of the results and contributed to the drafting of the manuscript. MC substantially contributed to the data analysis, preparation and interpretation of the results, and review of the manuscript. RC contributed to the study design, data collection and its monitoring and interpretation of the results and collaborated in the drafting of the manuscript. XC substantially contributed to the conception and design of the study and interpretation of the results and participated in the drafting of the manuscript. All the authors have reviewed and approved the final version of the manuscript.

## Authors’ information

We wish to highlight some relevant information about the authors: PL is a neurologist with a Master’s in Public Health, and was the main researcher of the PISCIS project, a community-based study of the incidence of stroke in Chile, whose results were published in indexed journals. XC is and PhD with vast experience in research and health service systems. LH has a Master’s in Public Health, is a Ph.D. candidate in Biomedicine and works at the School of Public Health in the *Universidad Mayor of Chile*.

## Pre-publication history

The pre-publication history for this paper can be accessed here:

http://www.biomedcentral.com/1471-2377/13/23/prepub
